# Anti-Inflammatory Therapies in Acute Coronary Syndromes—A Review of Immunological, Genetic, and Clinical Challenges for Precision Medicine

**DOI:** 10.3390/jcm15114143

**Published:** 2026-05-27

**Authors:** Mateusz Dudek, Natalia Górniak, Michał Ostrowski, Aleksandra Złotowska, Piotr Gajewski

**Affiliations:** 1 Student Scientific Group of Heart Diseases, Wroclaw Medical University, 50-556 Wrocław, Poland; natalia.gorniak@student.umw.edu.pl (N.G.); michal.ostrowski@student.umw.edu.pl (M.O.); aleksandra.zlotowska@student.umw.edu.pl (A.Z.); 2 Institute of Heart Diseases, Wroclaw Medical University, 50-556 Wrocław, Poland; p.gajewski@umw.edu.pl

**Keywords:** acute coronary syndromes, residual inflammatory risk, NLRP3 inflammasome, CHIP, precision cardiology

## Abstract

**Background:** Despite significant progress in management of acute coronary syndromes (ACSs), they continue to be a major cause of death worldwide due to residual inflammatory risk (RIR). **Aim:** This study reviews existing clinical evidence for anti-inflammatory therapies in coronary heart disease (CHD) and assesses precision medicine in classifying patients from clinical, immunological, and genetic perspectives. **Results:** Large clinical trials confirm the inflammatory hypothesis of atherosclerosis. Therapies targeted at the specific NLRP3 inflammasome/interleukin-1β (IL-1β)/interleukin-6 (IL-6) pathway reduce major adverse cardiovascular events (MACEs), while broad immunosuppression fails. This highlights the need for molecular specificity. Precision cardiology aims to identify high-risk inflammatory phenotypes through clonal hematopoiesis of indeterminate potential (CHIP). Mutations in genes such as *TET2* and *ASXL1* lead to macrophage hyperreactivity and increased plaque vulnerability. Available data suggest that the effectiveness of immunomodulatory treatment strongly depends on timing. Starting therapy early with SGLT2 inhibitors (SGLT2is) or agents that target temporarily activated receptors like P2Y11 seems to be essential for managing harmful inflammation while supporting myocardial repair. **Conclusions:** Precision cardiology aims to integrate targeted anti-inflammatory therapies with established clinical markers, while future pathways may incorporate advanced immunophenotyping and genetic risk assessment as they undergo clinical validation.

## 1. Introduction

Myocardial infarction (MI) is one of the leading causes of death worldwide. Despite significant advancements in cardiology, the mortality and morbidity associated with cardiovascular diseases (CVDs), particularly acute coronary syndromes (ACSs), remain high. Modern treatments, which include pharmacotherapy and revascularization, can reduce MI mortality. However, complications such as adverse cardiac remodeling or heart failure limit the benefits of modern standards of care [1,2]. Current pharmacological management, which relies on aggressive low-density lipoprotein cholesterol (LDL-C) lowering, is effective, but many patients still experience repeat cardiovascular events. Thus, it is justified to explore mechanisms that would allow for further risk reduction [3]. Scientific evidence indicates that inflammation plays a significant role in atherogenesis. Therefore, the inflammatory marker high-sensitivity C-reactive protein (hs-CRP) is a reliable predictor of cardiovascular risk, often proving more potent than LDL-C levels in patients receiving statin therapy. Its level enables a precise estimation of residual inflammatory risk (RIR) [4,5,6]. This connection is particularly clear in specific groups, such as patients with chronic kidney disease (CKD), suggesting the need to identify appropriate therapeutic agents aimed specifically at reducing the inflammatory process [7]. Atherosclerosis is a chronic inflammatory disease. Inflammation affects all phases of atherogenesis, inducing cell adhesion, lesion expansion, matrix and collagen degradation, and smooth muscle cell proliferation, while also increasing platelet activity and the risk of thrombosis. An intensified inflammatory response promotes plaque destabilization and rupture, leading to acute ischemia and myocardial necrosis [8,9]. The NLRP3 (NLR family pyrin domain containing 3) inflammasome has a significant function in the inflammation process. It is activated by, among other factors, oxidative stress, ischemia, cholesterol crystals, or mitochondrial dysfunction. This leads to the release of pro-inflammatory interleukin-1β (IL-1β), which induces the production of interleukin-6 (IL-6) [2,10,11]. IL-6 is considered the primary messenger in atherogenesis [12]. In light of the above, beyond hs-CRP, increasing attention is being paid to other potential indicators, such as the neutrophil-to-lymphocyte ratio (NLR), which reflects myeloid lineage activity and correlates with residual risk [13]. The CANTOS trial confirmed that selective inhibition of IL-1β using canakinumab reduces the risk of major adverse cardiovascular events (MACEs). One of the recorded side effects of this therapy is an increase in the incidence of fatal infections [4]. Non-specific anti-inflammatory treatment with colchicine in the COLCOT trial also demonstrated benefits in MACE reduction, and more recent data indicate that certain patient groups derive greater benefits than others, suggesting the need for further research into targeted treatment [14,15]. However, recent reports have suggested that the efficacy of this therapy may decrease over extended follow-up [16]. The CIRT trial demonstrated the ineffectiveness of methotrexate in reducing the incidence of MACE. This agent did not reduce the levels of IL-1β, IL-6, or hs-CRP. The outcome of this study proves the urgency of therapy targeting a specific inflammatory pathway rather than non-selective immunosuppression [17]. Following the conflicting results of previous therapies, the RESCUE trial focused on directly inhibiting IL-6. The phase-2 study proved the efficacy of ziltivekimab in reducing inflammatory biomarkers, specifically hs-CRP and NLR, without exposing patients to severe infections, as observed with canakinumab, potentially making it a safer alternative [12,13,18]. Currently, considering the varied results of clinical trials, it is evident that a universal strategy is insufficient and represents a significant challenge. Studies such as CIRT, which used methotrexate, confirm that a more targeted approach tailored to individual patients is necessary [17]. Accordingly, a patient identification system is required to select the most appropriate treatment. Various methods exist for tailoring therapy to patients. One of these involves a specific clinical profile, as in the case of the RESCUE trial, where effective treatment was administered to patients with chronic kidney disease (CKD) and elevated hs-CRP [12]. Another approach involves using a biomarker profile for treatment selection, for instance, by using the NLR [13]. Furthermore, a promising strategy involves treating patients based on an analysis of their genetic predispositions, specifically somatic mutations associated with clonal hematopoiesis of indeterminate potential (CHIP), such as variants in the Ten-Eleven Translocation 2 (*TET2*) and DNA Methyltransferase 3 Alpha (*DNMT3A*) genes, which modify the response to anti-inflammatory treatment [19]. The integration of the above-mentioned patient stratification systems appears to be the optimal solution. However, their current absence is a major barrier to implementing the most beneficial therapy.

This study provides an integrative framework by bridging molecular immunology with the latest genetic insights into CHIP and recent clinical trial evidence. We summarize existing data and propose a precision medicine algorithm that accounts for patient-specific inflammatory phenotypes and optimal therapeutic timing. By integrating clinical, immunological and genetic perspectives, this paper aims to guide clinicians on which patients benefit most from anti-inflammatory treatment.

## 2. Methodology and Search Strategy

This article was conducted as a comprehensive narrative expert review. A literature search was performed across the PubMed and Google Scholar databases in March 2026 without any additional filters. The search strategy contained a combination of medical subject headings (MeSHs) and relevant keywords, including: “acute coronary syndrome”, “NLRP3 inflammasome”, “residual inflammatory risk”, “CHIP” and “precision cardiology”. We prioritized high-impact clinical trials, meta-analyses and mechanistic reviews that provided insights into the molecular pathways of atherothrombosis. Studies were selected based on their clinical relevance and the currency of the clinical evidence.

## 3. Immunological Basis of Inflammation in Acute Coronary Syndromes

The relationship between inflammation and ACS is complex and not fully understood. Atherosclerosis is a chronic inflammatory disease, and its acute and chronic complications constitute the primary cause of ACS. Plaque rupture, as one of the acute complications of atherosclerosis, leads to thrombus formation and a reduction in the blood supply to the myocardium, which may contribute to the occurrence of an MI. Such a scenario accounts for the majority of ACS cases [9,20]. Unstable plaques, composed of a lipid-rich core and foam cells covered by a thin fibrous cap, are most susceptible to rupture [21]. The cause of plaque rupture is the exacerbation of inflammation within it. Myocardial necrosis resulting from this incident constitutes another source of inflammatory response in the body [9]. Both innate and adaptive immune responses are associated with the development of inflammation in the atherosclerotic plaque. Monocytes appear in the plaque, recruited by molecules such as selectins, monocyte chemoattractant protein-1 (MCP-1), and vascular cell adhesion molecule-1 (VCAM-1) located on endothelial cells. Monocytes differentiate into M1-type macrophages, that is, those with a pro-inflammatory phenotype [22]. Accumulating macrophages produce enzymes, such as matrix metalloproteinases (MMPs) or cathepsins, which degrade the extracellular matrix. Consequently, the balance between the levels of these enzymes and their inhibitors is disrupted, which promotes plaque rupture [23]. T lymphocytes, particularly Th1, also participate in this process by stimulating the synthesis of interferon gamma (IFN-γ), which inhibits the proliferation of vascular smooth muscle cells and influences the direction of macrophage proliferation [24].

An essential element of the inflammation contributing to plaque rupture is also the NLRP3 inflammasome, the activation of which causes the release of further pro-inflammatory cytokines and an inflammatory response in the blood vessel wall, contributing to the progressive development of atherosclerosis [25]. Studies have shown that the plasma level of the NLRP3 inflammasome in patients with ACS is associated with the severity of the disease course; when high, it indicates a poorer short-term prognosis [26]. The NLRP3 inflammasome is a cytoplasmic complex formed from several proteins in response to pathogen-associated molecular patterns (PAMPs) or damage-associated molecular patterns (DAMPs). This complex forms when the NLRP3 receptor, belonging to the group of pattern recognition receptors (PRRs), recognizes the aforementioned molecular patterns [27]. Two signals are required for NLRP3 inflammasome activation. The priming signal consists of increasing the expression of NLRP3 and pro-IL-1β proteins through the activation of the NF-kB pathway. It begins, among others, in response to ligands for toll-like receptors (TLRs) or NOD-like receptors (NLRs). In contrast, the second signal (activation signal) leads to the formation of the NLRP3 inflammasome complex through mechanisms such as overproduction of reactive oxygen species (ROS), K^+^ efflux, endoplasmic reticulum stress, Na^+^ influx with simultaneous Cl^−^ efflux, mitochondrial dysfunction, Ca^2+^ signaling, or lysosomal destabilization. These mechanisms are significant in the progressive atherosclerotic process [11,28].

Additionally, in the context of atherosclerosis and ACS, it is significant that cholesterol crystals (CCs) participate in NLRP3 inflammasome activation. Their presence in the atherosclerotic plaque indicates a high degree of disease advancement and an increased risk of plaque rupture. Studies have shown that atherosclerotic lesions containing CCs had more features of an unstable plaque, such as the presence of calcifications, macrophage accumulation, or thrombus formation. Patients with ACS and CCs in culprit lesions presented higher NLRP3 expression in macrophages and higher levels of cytokines activated by the inflammasome: IL-1β and interleukin-18 (IL-18) [29]. Similar results were obtained in other studies, which additionally showed that CCs activate the complement system locally and systemically, and the interaction between CCs and complement may influence NLRP3 inflammasome activation [30]. CCs, like other solid particles, damage lysosomes after phagocytosis, which leads to lysosomal destabilization and the leakage of their contents into the cytosol. It is not fully explained which mechanism links lysosomal breakdown with NLRP3 inflammasome activation. However, these processes appear to be significantly interconnected [28].

**Figure 1:**
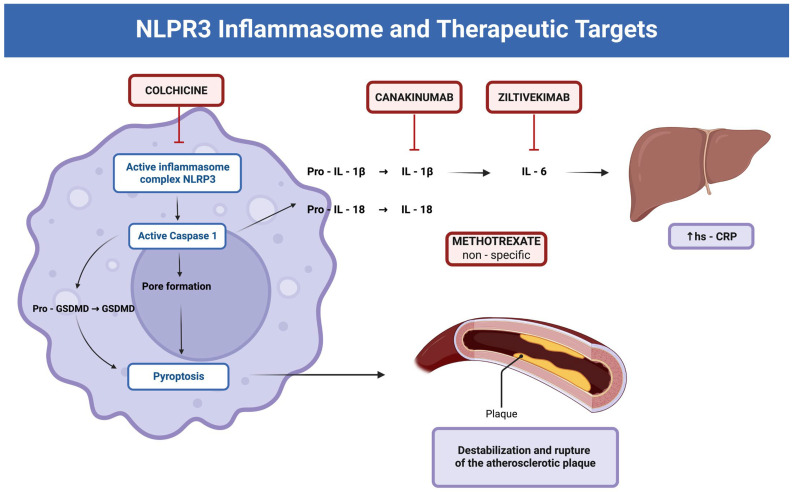
The NLRP3 inflammasome signaling pathway and therapeutic targets in coronary artery disease. The schematic shows how the NLRP3 inflammasome complex activates inside cells. This leads to the activation of caspase-1, which then cleaves gasdermin D (GSDMD). As a result, pores form, causing cell death through pyroptosis and further inducing destabilization and disruption of atherosclerotic plaques. The following signaling triggers the maturation of pro-inflammatory cytokines like IL-1β and IL-18. IL-1β induces the production of IL-6, which then stimulates the liver to produce acute-phase proteins, particularly hs-CRP. The red bars indicate the molecular targets for colchicine (NLRP3), canakinumab (IL-1β), and ziltivekimab (IL-6). Methotrexate is noted as a non-specific anti-inflammatory agent with a different mechanism of action.

The NLRP3 inflammasome complex includes 3 parts: NLRP3, also known as cryopyrin, apoptosis-associated speck-like protein containing a CARD (ASC), and caspase-1 [31]. NLRP3 serves as a sensor and consists of three parts: the N-terminal pyrin domain (PYD), central NBD-containing ATPase domain (NACHT), and C-terminal leucine-rich repeats (LRRs). The ASC protein, being an adapter, is composed of two parts: the N-terminal PYD and the C-terminal caspase recruitment domain (CARD). Caspase-1, serving as the inflammasome effector, consists of three parts: the N-terminal CARD and two catalytic domains: the larger p20 and the smaller p10 [32]. After inflammasome activation, the NACHT domain connects the sensor subunits of NLRP3 together. In this way, a platform is created for the oligomerization of the ASC protein, which occurs through the interaction of its PYD with the PYD domain contained in the NLRP3 sensor. Thanks to this, the recruitment of pro-caspase-1 and its activation becomes possible, because it is the ASC protein that contains the corresponding CARD. Thus, an interaction occurs between the CARDs of the ASC protein and pro-caspase-1, which effectively causes the autocatalytic conversion of pro-caspase-1 to caspase-1, thereby initiating its proteolytic activity. Active caspase-1 induces the conversion of pro-IL-18 and pro-IL-1β into their biologically mature forms and the cleavage of gasdermin D (GSDMD) [10]. As a result of GSDMD activation, pores are formed in the cell membrane through which IL-1β and IL-18, as well as active ASC protein specks, are released from the cell, in which, in turn, pyroptosis begins (Figure 1). Extracellular ASC protein specks can be detected in the plasma of patients with conditions in which NLRP3 inflammasome activation occurs, such as ACS. However, currently used methods detect the ASC protein itself, and it also cooperates with other receptors besides NLRP3, making it impossible to determine specifically which inflammatory process is occurring in the body [33,34].

Cytokines IL-1β and IL-18, resulting from NLRP3 inflammasome activation, along with interleukin-1α (IL-1α), among others, are part of the IL-1 cytokine group. IL-1α in an inactive form is present in all mesenchymal cells, including myocardial cells. After release resulting from necrosis, it becomes active and can induce the production of IL-1β. Both IL-1β and IL-1α disrupt vasodilation, intensify oxidative stress, and increase the production of procoagulant mediators, thereby contributing to the development of thrombosis on the basis of atherosclerosis and, consequently, ACS [35]. In the context of these disorders, it is also significant that LDL-C, the level of which is elevated in patients with increased cardiovascular risk and MI, contributes to the production of IL-1β. It stimulates IL-1β production dependent on the lectin-like oxidized low-density lipoprotein receptor-1 (LOX-1). As a result of the stimulation of this receptor, the transcription factor NF-kB and the NLRP3 inflammasome are activated, leading to the initiation of caspase-1 action and the conversion of pro-IL-1β to its active form [36].

Although the presence of IL-1β has been confirmed in atherosclerotic plaques and the neutralization of this cytokine’s action by appropriate drugs reduces the risk of cardiovascular events in at-risk groups, its level in their blood is not significantly elevated [35]. A more promising marker for assessing the risk of cardiovascular events appears to be the second cytokine resulting from NLRP3 inflammasome activation, namely IL-18. Studies have shown that patients diagnosed with ACS presented an elevated level of IL-18, which was also associated with an increased risk of adverse cardiovascular events in these patients [37]. In the inflammatory process, IL-18 induces the production of cytokines, such as IFN-ɣ, and activates monocytes to differentiate into foam cells, which promotes the progressive development of atherosclerosis [38].

The IL-1 group of cytokines also systemically stimulates the production of the cytokine IL-6. IL-6 stimulates hepatocytes to produce acute-phase proteins, such as fibrinogen, plasminogen activator inhibitor-1 (PAI-1), or C-reactive protein (CRP). Fibrinogen participates in thrombus formation, while PAI-1 inhibits fibrinolysis; thus, they collectively favor the development of thrombosis against the background of atherosclerosis and the occurrence of ACS. In contrast, CRP is a significant marker of inflammation [39]. Its level in the blood can be determined using a test called hs-CRP. Studies have shown that a slightly elevated level of hs-CRP (between 10 and 15 mg/L) was associated with a higher risk of death in patients with suspected ACS. Therefore, hs-CRP, alongside the standardly used troponin, could potentially be a significant marker for use in clinical practice in patients with ACS [40].

Recent studies suggest that an important prognostic marker in ACS may also be the advanced lung cancer inflammation index (ALI). It is an index calculated from body mass index (BMI), serum albumin levels and NLR (BMI × albumin/NLR). Lower ALI values have been associated with higher mortality among patients with ST-elevation myocardial infarction (STEMI). Additionally, ALI appears to perform better than NLR alone, as it takes into account not only the presence of inflammation in the body, but also the nutritional status of the patient [41].

NLRP3 inflammasome activation, besides the release of IL-1 group cytokines, leads to the initiation of pyroptosis in the cell. Pyroptosis is a type of programmed cell death during which nuclear condensation, DNA fragmentation, and the formation of the previously mentioned pores in the cell membrane, involving GSDMD, occur. It affects cells essential in the pathophysiology of atherosclerosis—endothelial cells, macrophages, and vascular smooth muscle cells. As a result of endothelial cell death, its dysfunction progresses, favoring the formation of atherosclerotic plaques. Macrophages, after death, form the necrotic core of the plaques and contribute to the thinning of their fibrous cap, making the plaques adopt an unstable phenotype. Macrophage pyroptosis is induced by oxidized forms of LDL (ox-LDL) accumulating in vessels during the course of atherosclerosis, and the process itself involves interferon regulatory factor 1 (IRF-1), which activates caspase-1, crucial in the course of this type of death. As a result of the interactions on the mentioned cells, pyroptosis may accelerate the progression of atherosclerosis, favor the rupture of unstable atherosclerotic plaques, and lead to the occurrence of ACS. Furthermore, pyroptosis occurs in the cardiomyocytes themselves, leading to a reduction in the number of these cells, impairment of myocardial contractility, and adverse cardiac remodeling [42,43].

## 4. Inflammation in Stable Coronary Artery Disease

The common foundation for both stable CAD and ACS is inflammation, which determines atherosclerotic plaque stability and its susceptibility to rupture. The consequences of plaque rupture, whether it is a fibrous cap rupture or superficial erosion, are dependent on inflammation [44]. It serves as the driving force for atherosclerosis and the transition from stable CAD to ACS. The systemic inflammatory response determines the fate of the atherosclerotic plaque, which may grow slowly, causing stable disease, or lead to sudden thrombosis and ACS [45]. The following studies on stable CAD have shown that reducing inflammation is correlated with a decrease in cardiovascular events. Anti-inflammatory therapies have demonstrated benefits in the case of chronic treatment, as evidenced by the LoDoCo2 and CANTOS trials.

### 4.1. CANTOS Trial—Canakinumab

The first study to confirm the inflammatory hypothesis was the randomized, placebo-controlled, double-blind CANTOS trial, which was conducted in 2011–2017. It involved 10,061 patients after an MI with hs-CRP > 2 mg/L [4]. Anti-inflammatory therapy inhibiting IL-1β signaling using canakinumab at a dose of 150 mg every 3 months was associated with a significantly lower rate of recurrent cardiovascular events, and the incidence of MACE was reduced by 15% (HR 0.85, 95% CI 0.74–0.98). The use of canakinumab resulted in a reduction in hs-CRP and IL-6 by 35–40%. An adverse effect of canakinumab therapy was a small but statistically significant increase in the number of deaths caused by infection and sepsis [4]. A particular benefit was noted in individuals with CKD, where only hs-CRP and IL-6 predicted MACE. LDL-C and HDL-C (high-density lipoprotein cholesterol) were not significant [7]. This may indicate the need to target the treatment of inflammation among specific patient groups, such as those at higher risk of inflammation. However, despite the reduced frequency of cardiovascular events following IL-1β blockade, canakinumab was not approved for use in secondary cardiovascular prevention. The risk of severe infections, the lack of a clear dose–response, the drug’s injectable form, and an estimated annual cost of $200,000 limit the widespread use of this preparation [46].

### 4.2. LoDoCo and LoDoCo2 Trials—Colchicine

Another key clinical trial was LoDoCo2, which resulted in a 31% lower relative risk of cardiovascular death, spontaneous MI, ischemic stroke, or ischemia-driven coronary revascularization. Importantly, in the earlier LoDoCo trial, despite using the lowest available dose of colchicine, 11% of patients discontinued therapy prematurely due to intestinal intolerance [47]. In the LoDoCo2 trial, this percentage was higher, with as many as 15.4% of patients included in the run-in phase not undergoing randomization due to gastrointestinal (GI) complaints [48]. Differences emerged in the magnitude of the treatment effect between the Australian and Dutch populations, which researchers attribute to the difference in observation time [49]. The protective effect of the drug was consistent across all risk groups, which means that in the case of chronic coronary disease, low-risk patients also derive significant benefit from the therapy [50]. In the post-randomization phase of the LoDoCo2 trial, low-dose colchicine slightly increased the incidence of mild myalgia compared to the placebo, but this rarely resulted in treatment discontinuation [51]. Early intolerance to low-dose colchicine is dose-dependent, occurs more frequently in women and obese patients and is primarily limited to mild, self-limiting GI symptoms. Among patients who tolerate the initiation of therapy, long-term tolerability appears acceptable [52]; however, real-world safety strictly requires avoiding drug–drug interactions and routinely monitoring renal function. It is important to highlight potential concerns regarding non-cardiovascular mortality observed in trials such as LoDoCo2. However, this trend has not been observed in other cardiovascular disease studies, and subsequent meta-analyses confirm that colchicine therapy does not affect non-cardiovascular mortality. In the context of long-term, high-dose administration in patients with advanced renal or hepatic impairment, other potentially severe adverse effects, such as chronic cytopenias and neuromyopathies, may occur, though these were likely associated with sustained serum concentrations above 3.0 μg/L [52]. Due to its narrow therapeutic index, colchicine toxicity can easily occur, particularly when patients develop excessive serum concentrations. This accumulation often results from a combination of excessive dosing, drug interactions, and impaired renal or hepatic function. The medications that interact with colchicine include antiarrhythmics (such as amiodarone or propafenone), calcium-channel blockers (CCBs), statins, fibrates, macrolide antibiotics and calcineurin inhibitors. Once toxicity manifests, treatment is highly challenging because the subsequent damage to the kidneys and liver further compromises the patient’s capacity to eliminate the drug from the body. Renal impairment predisposes patients to colchicine toxicity and hepatic dysfunction can similarly elevate the risk of adverse drug events due to drug–drug interactions [53]. Additionally, in patients with chronic coronary disease, low-dose colchicine showed no drug–drug interactions with vitamin K antagonists (VKAs), indicating that additional International Normalized Ratio (INR) monitoring is not routinely required [54]. Although colchicine does not affect cholesterol levels, the benefits of its use are comparable to, or even greater than, those of modern LDL-C-lowering drugs, such as PCSK9 inhibitors or ezetimibe [55,56].

### 4.3. CIRT Trial—Methotrexate

The third important study in the context of the inflammatory hypothesis is CIRT, focusing on low-dose methotrexate, which is an inexpensive, effective treatment for certain inflammatory conditions. The study was terminated prematurely because doses of 15 to 20 mg did not result in a reduction in IL-1β, IL-6, or CRP levels and did not reduce the number of cardiovascular events. This probably results from the fact that the CIRT study, unlike CANTOS, did not include screening for CRP levels. The median in CIRT participants was significantly lower (1.6 mg/L) than in the CANTOS study (4.2 mg/L) [17]. Combined with data from CIRT, where methotrexate neither reduced the frequency of events nor reduced the concentration of IL-6, this suggests that drugs that do not lower IL-6 levels will not be effective in protecting against atherosclerosis [12]. Low-dose methotrexate has proven ineffective due to its non-selective mechanism of action. Furthermore, methotrexate can be highly harmful to patients with renal impairment since over 90% of the drug is excreted through the kidneys, requiring strict monitoring, dose reduction or complete discontinuation. This clinical challenge is compounded by concerns regarding hematologic and GI toxicities [57,58]. Additionally, higher levels of methotrexate metabolites (polyglutamates) are associated with a higher risk of anemia, and a level >134 nmol/L with liver abnormalities. Interestingly, in the CIRT study, the group receiving low doses of methotrexate more frequently had leukopenia compared to the placebo group, and the study “Adverse effects related to methotrexate polyglutamate levels: adjudicated results from the cardiovascular inflammation reduction trial” showed no correlation between methotrexate polyglutamate levels and leukopenia. This shows that the drug carries a significant risk of adverse effects in patients not treated for rheumatological reasons, and monitoring toxicity would pose an additional difficulty [59]. Based on CIRT, it appears that methotrexate, unlike canakinumab (CANTOS) and colchicine (COLCOT), acts through a different pathway than the NLRP3–IL-1β inflammasome [60,61].

## 5. Inflammation in Acute Coronary Syndromes

The direct cause of ACS is sudden injury to the atherosclerotic plaque, which exposes the highly thrombogenic core. The resulting thrombus rapidly blocks blood flow in the coronary artery, leading to myocardial ischemia and symptoms of ACS. High concentrations of hs-CRP are associated with a more frequent occurrence of ischemia [62,63].

### 5.1. COLCOT and COLOCT Trials—Colchicine

The study focusing on the use of colchicine immediately after an infarction (<30 days) is COLCOT. Daily use of a low dose (0.5 mg) significantly reduced the risk of serious cardiovascular complications such as recurrent infarction, stroke, or hospitalization for angina (5.5% vs. 7.1% in the placebo group) [14]. Data from COLCOT and LoDoCo suggest that the reduction in inflammation may be a key element in the clinical efficacy of low-dose colchicine after MI, considering the reduction in hs-CRP levels below the low-risk threshold of ≤1 mg/L [64]. Although the most frequently observed adverse effects were GI, their overall incidence was similar in both groups. Conversely, pneumonia occurred more frequently in the group of patients treated with colchicine (0.9% vs. 0.4%). Researchers indicate that colchicine, unlike canakinumab, did not increase the incidence of septic shock. A limitation of the study was the relatively short observation time of 23 months, which did not allow for the evaluation of long-term colchicine treatment [14]. An economic analysis based on the Canadian healthcare system showed that colchicine, as an addition to standard therapy after MI, reduced healthcare costs during the study by 47% and over the entire lifespan by 69%, and the corresponding increase in the quality-adjusted life year (QALY) index was 0.04 and 2.87. Cost-effectiveness results from the reduction in the number of expensive clinical events as well as the low price of this drug [65]. Drug tolerance may be genetically determined, as the intergenic variant rs6916345 present on chromosome 6 is associated with a more frequent occurrence of diarrhea and was previously linked to Crohn’s disease. Furthermore, colchicine appears not to yield significant cardiovascular benefits, such as preventing future MI, in individuals with specific polymorphisms [66]. In the COLOCT study, after examining patients with ACS and lipid-rich atherosclerotic plaque, it was shown that the use of colchicine for one year significantly strengthened the structure of the atherosclerotic plaque, increasing fibrous cap thickness by an average of 34.2 μm [67]. The therapy reduced the mean lipid arc by 10.5° and resulted in a reduction in macrophage infiltration, which is directly responsible for the degradation of the fibrous cap, potentially leading to plaque rupture. The presence of their infiltrates drastically increases the risk of death or recurrent MI. This is associated with an increased risk of recurrent adverse cardiac events after ACS [68,69]. Moreover, the reduction in inflammation was particularly beneficial in patients with coronary artery disease and well-controlled LDL-C ≤ 70 mg/dL. This discovery supports the thesis that anti-inflammatory therapy by adding colchicine to standard therapy has a beneficial effect on atherosclerotic plaque stabilization in patients with ACS [67]. Despite its efficacy, colchicine uptake remains limited due to safety concerns regarding severe toxicity, the need for dose adjustment for kidney dysfunction, and interactions with drugs [70]. However, data from the COLCORONA trial suggest that the concomitant use of low-dose colchicine with statins (such as atorvastatin and rosuvastatin) or CCBs did not significantly increase the risk of GI or overall adverse events [71]. Analysis based on the FAERS database indicates that co-administration of colchicine and statins may increase the risk of rhabdomyolysis, particularly when combined with atorvastatin or rosuvastatin [72]. The authors suggest that the increase in adverse effects due to the combination of colchicine and statins occurs because the competition for the same metabolic enzymes and efflux pumps between colchicine and lipophilic statins may result in elevated plasma levels of these drugs [72]. To explain these clinical differences between safety registries and controlled trials, the authors argue that the incidence of colchicine drug–drug interactions is lower in low-dose clinical trials following MI compared to real-world clinical practice. In general settings, patients may receive higher doses of colchicine, are not as thoroughly screened for risk factors and concomitant therapies and may not be as closely monitored for adverse effects [53]. On the other hand, contemporary multi-center evidence strongly contradicts these concerns. Cumulative data from randomized controlled trials involving over 30,000 patient-years demonstrate that in patients with adequate renal function, the incidence of myotoxicity or serious drug–drug interactions with statins is rare and no more frequent than with a placebo. The discrepancy exists because the FAERS database does not record information about the renal function or the dose or time of the last dose of colchicine and does not ascribe causality of myotoxicity to colchicine. Thus, concerns over myotoxicity should not preclude the use of low-dose colchicine in statin-treated cardiovascular patients, as long as significant renal or hepatic dysfunction is ruled out. Additionally, no evidence of hepatotoxicity was observed in either the LoDoCo2 or COLCOT trials, in which virtually all participants were taking statin therapy. Fatal drug interactions with colchicine only happened when it was taken together with clarithromycin or cyclosporine at doses of 1.5 mg or higher, leading to pancytopenia [51].

### 5.2. CLEAR-SYNERGY Trial—Colchicine

Results in the acute phase are not uniform, as evidenced by the results of CLEAR-SYNERGY, which contrast with, for example, COLCOT. Although colchicine lowered the CRP level by −1.28 mg/L, no statistically significant reduction in cardiovascular events was demonstrated after the use of colchicine (9.1% vs. 9.3%). Notably, CLEAR-SYNERGY demonstrated CRP reduction without a corresponding decrease in MACE after MI. It is worth emphasizing that participants initially received 0.5 mg of the drug twice daily or a placebo. However, as a result of an increased dropout rate, the dose was reduced to 0.5 mg once daily [16]. In this study, diarrhea also occurred more frequently in the colchicine group (10.2% vs. 6.6%), but no clear difference in the number of serious infections was observed between the groups. Researchers recorded 649 primary endpoints and argue that studies with >600 are rarely erroneous. They claim that subgroup analyses do not suggest that the COVID-19 pandemic influenced the results and that the reduced CRP level and diarrhea testify to the biological effect of colchicine. A limitation of the study was the insufficient representation of women and participants of races other than white [16]. Another analysis also points to the results in the CLEAR-SYNERGY study, but the authors claim that the failure is related to high treatment discontinuation rates, reduced medical adherence, and disruptions in the studies during the COVID-19 pandemic [73].

## 6. Special Patient Groups and New Research Directions

In the case of the acute phase of coronary artery disease, the results are not as uniform as in the case of studies related to stable coronary artery disease.

### 6.1. RESCUE Trial—Ziltivekimab

In the CANTOS trial, participants with CKD and elevated IL-6 levels benefit most from targeted anti-inflammatory therapy. However, colchicine is contraindicated in this group because it is excreted by the kidneys. Utilizing ziltivekimab in the RESCUE study in patients with moderate to severe CKD and hs-CRP > 2 mg/L, the median CRP decreased by 88% in the 15 mg group and by 92% in the 30 mg group. The mean dose of 15 mg was nearly as effective as the 30 mg dose. Compared to the placebo group, triglyceride concentrations increased [12]. These results were confirmed by the RESCUE-2 study in Japan, although the participants were generally healthier than the participants in the US study; a smaller percentage took statins, had diabetes, or ASCVD. RESCUE-2 participants also had lower median hs-CRP levels at the start of the study. No significant differences in the frequency of infections were found [18]. The scale of hs-CRP reduction in the RESCUE study was approximately twice as large as that obtained in the CANTOS study. A post hoc analysis of RESCUE shows that ziltivekimab is associated with a lower NLR, which independently predicts atherosclerotic events and is a potential biomarker of residual inflammatory risk. After 12 weeks, the estimated difference compared to the placebo was −15.3% and −23.6% for the 15 mg and 30 mg doses, respectively. In the event of introducing ziltivekimab into clinical practice, it will be possible to assess treatment effectiveness thanks to NLR, which is easily available in routine blood counts [13].

### 6.2. ZEUS Trial—Ziltivekimab

Despite the small population in the RESCUE study and short study duration, the results qualified ziltivekimab for the long-term ZEUS trial [74]. Research results indicate that direct inhibition of IL-6 may prevent the formation of arterial thrombi. A model was developed using collagen exposure in the basement membrane, similar to what occurs after atherosclerotic plaque erosion. In mice with chronic low-grade inflammation treated with an anti-IL-6 monoclonal antibody, thrombus formation was significantly reduced, with a simultaneous lack of effect on plasma coagulation factors. The effect was attributed to weakened platelet activation by collagen, which was confirmed by ex vivo stimulation with collagen-rP. A similar effect on human platelets was confirmed by the results of therapy with biosimilar ziltivekimab in 15 patients after PCI with very high cardiovascular risk. The aforementioned study promotes the potential use of ziltivekimab to reduce residual risk and prevent major atherothrombotic events, as the vast majority of patients with very high cardiovascular risk receive antiplatelet drugs for this purpose [75]. Furthermore, a comprehensive genetic analysis by Zhang et al. [76] mimicking the long-term effects of ziltivekimab concluded that IL-6 inhibition reduces cardiovascular risk without major unexpected safety concerns. Nevertheless, their models revealed potential warning signals for migraine, open-angle glaucoma, and pregnancy-related maternal hemorrhage [76]. These genetic models suggest that long-term IL-6 inhibition may be associated with a higher risk of these conditions, along with reductions in white blood cell and platelet counts, though these findings remain to be validated by long-term clinical data using the actual therapeutic agent. Mechanistically, the data showed a clear link between IL-6 downregulation and increased triglyceride content, particularly within HDL-C particles [76]. This matches earlier clinical reports where ziltivekimab was explicitly connected to higher circulating triglyceride levels [18]. The ZEUS trial design evaluates the effectiveness of ziltivekimab in reducing cardiovascular incidents in patients with atherosclerosis, CKD, and hs-CRP ≥ 2 mg/L. In 6376 patients under observation, the median hs-CRP level was 4.5 mg/L, and the median IL-6 level was 4.9 pg/mL. The authors argue that inhibiting IL-6 is crucial for atherosclerotic plaque development and may be an effective therapeutic option for patients with impaired renal function [77]. The use of ziltivekimab and anti-inflammatory therapies was justified by the fact that IL-6 drives inflammatory processes in the body, the symptom of which is high hs-CRP produced by the liver. hs-CRP was presented as a signal of chronic systemic inflammation that drives the activation of cells in the atherosclerotic plaque, leading to its rupture. It explains that on the basis of trained immunity, innate immune cells have a tendency to produce IL-1β and IL-6, intensifying CRP production and ASCVD development. Trained immunity is induced by infection, hypercholesterolemia, hyperglycemia, or a Western diet. A correlation between monocyte behavior and increased concentrations of pro-inflammatory cytokines with hs-CRP was presented in patients with unstable angina. The author believes that hs-CRP may reflect the degree of immune system reprogramming, and in the future, a form of treatment could be epigenetic modifications of immune cells so that they block the production of inflammatory cytokines [78].

### 6.3. Anakinra

The VCU-ART and VCU-ART2 studies on anakinra, which is an IL-1 antagonist, showed a decrease in the acute inflammatory reaction. It appears that IL-1 blockade with anakinra for 2 weeks has a neutral effect on recurrent ischemic events but may prevent new cases of heart failure (HF) in the long-term perspective after a STEMI [79]. However, in the MRC-ILA-Heart study on 182 non-ST-elevation myocardial infarction (NSTEMI) patients whose chest pain occurred <48 h earlier, 100 mg of anakinra was administered subcutaneously. In this case, the drug did not improve clinical results and only lowered CRP. Adverse effects resulting from the treatment were most frequently pain and erythema at the injection site, occurring in approximately 20% of patients. Active or recurrent infections remain contraindications to therapy; additionally, long-term therapy requires periodic monitoring of the neutrophil count. On the other hand, the rapid half-life of anakinra is a key advantage in ACS as it allows for immediate treatment withdrawal if patients develop even mild symptoms of infection [80].

### 6.4. Dapansutrile

New research directions also focus on direct, oral NLRP3 inhibitors, such as dapansutrile. This approach aims to safely reduce IL-1β-mediated inflammation without the risk of systemic immunosuppression or infections associated with injectable monoclonal antibodies [81]. Dapansutrile has been evaluated in acute gout flare. The initial PODAGRA I dose-finding study showed preliminary evidence of safety and efficacy [81], while broader phase 2 data demonstrated significant hs-CRP reductions and safety at doses up to 1000 mg/day [82]. Currently, its potential is being further investigated in the multi-center PODAGRA II trial, which represents the first large, placebo-controlled study of an oral NLRP3-specific inhibitor [81]. However, there are no clinical outcome data for post-ACS prevention with dapansutrile. Therefore, its efficacy and safety remain unproven in cardiovascular trials, keeping it as a promising strategy in future cardiological immunomodulation therapies [82].

### 6.5. Summary

A meta-analysis of 23 randomized controlled trials (RCTs) suggests that in acute myocardial infarction (AMI), timing is important when making a clinical decision. Only therapy initiated within 24 h was effective [83]. In the case of colchicine, conversely, early intervention (up to 72 h) may reduce the risk of recurrence by 50% compared to later treatment [73]. The CLEAR-SYNERGY study did not demonstrate effectiveness, and a sub-analysis of COLCOT suggested benefits from early colchicine administration. Delayed immunomodulation may interfere with repair processes; in the MRC-ILA study, IL-1β inhibition was initiated up to 48 h after the onset of symptoms and was associated with a higher frequency of adverse effects [80]. It is important that colchicine or IL-1β inhibitors do not significantly increase the risk of infection, suggesting that correctly used therapies can effectively mitigate inflammation without weakening the defense mechanisms of the immune system [83]. It should be noted that prospective studies are based mainly on a stable population, in which small elevations in hs-CRP appear to predict a continuous risk of MI and other cardiovascular events, although this effect may be small [80]. Others, focusing specifically on the time of ACS, did not observe hs-CRP. Despite a small effect, hs-CRP helps predict the risk of infarction in the stable population; however, the authors note that when the patient is already undergoing ACS, hs-CRP then loses its predictive value [84]. The results of the clinical trials described above are summarized in Table 1 below. This overview shows which inflammatory mechanism was targeted by each drug and details the clinical outcomes of each study. Information regarding the pharmacological dosing regimens and administration protocols used in the aforementioned clinical trials is summarized in Table 2. A nuanced perspective is essential when addressing anti-inflammatory treatments, as the available clinical evidence remains highly inconsistent. While CANTOS [4] and COLCOT [14] showed favorable clinical outcomes, these results contrast with the neutral findings from CIRT [17] and CLEAR-SYNERGY [16]. As a result, the clinical utility of ziltivekimab remains supported primarily by biomarker data pending definitive outcome trials. The recently published baseline characteristics and design of the ZEUS trial support the link between chronic inflammation, cardiovascular disease and CKD. Specifically, lower kidney function correlates with elevated levels of IL-6 and hs-CRP [77]. Beyond clinical efficacy, several safety and implementation issues critically impact the practical application of these targeted therapies. These include an elevated infection risk, GI intolerance and treatment adherence challenges in real-world scenarios. Potential non-cardiovascular mortality concerns observed in trials like LoDoCo2, along with risks of renal dysfunction and drug–drug interactions, pose significant practical limitations to the routine care utilization of colchicine. Consequently, although low-dose colchicine has received Food and Drug Administration (FDA) approval for cardiovascular risk reduction, its adoption in international guidelines and everyday clinical practice remains relatively cautious [70]. Current literature on colchicine drug–drug interactions remains highly inconsistent, creating what researchers describe as a “jumble of confusion” with conflicting recommendations across various scientific sources [72]. While low-dose clinical trials report minimal risks, real-world data indicate that the concurrent use of colchicine and lipophilic statins can trigger severe myotoxicity due to competition for identical metabolic pathways and efflux pumps [72]. The authors criticize automatic colchicine dosage reductions due to high inter-patient variability, arguing that the common recommendation to reduce the dose when given with CYP3A4/P-gp inhibitors is “likely to result in colchicine toxicity in some patients and therapeutic failure in others”. Crucially, they emphasize that these concomitant combinations can trigger life-threatening drug–drug interactions, including pancytopenia, multiorgan failure and cardiac arrhythmias. Consequently, rather than attempting risky dose adjustments, the safest clinical approach to preventing life-threatening toxicities is to avoid these interacting combinations entirely whenever feasible [53]. To translate clinical trial data into a practical tool, Table 3 shows a patient-stratification framework for precision cardio-immunology. This approach directly links clinical, immunological and genetic patient profiles to targeted anti-inflammatory regimens. This allows clinicians to use precise secondary prevention. Importantly, while some of these targeted frameworks represent standard clinical care, others involve experimental agents or therapies not yet formally approved for cardiovascular prevention.

## 7. Genetics and Inflammatory Phenotyping—Elements of Precision Cardiology

### 7.1. Precision Medicine and the Necessity of Personalization

Meta-analyses prove that the efficacy of anti-inflammatory treatment in CAD depends mainly on targeting a specific pathway rather than using broad-spectrum anti-inflammatory drugs. Immunomodulatory drugs as a group do not statistically significantly reduce the risk of MACE, but specific therapies focusing on the NLRP3 inflammasome and interleukin pathway show efficacy in clinical trials. They reduce the risk of MACE and improve left ventricular ejection fraction (LVEF), especially with long-term use (>6 months) [90]. This shows the need for the precise identification of patients who would potentially respond best to the applied therapy. Inflammatory pathophysiology differs in acute states such as MI, where tissue necrosis and IL-1α release activate the inflammasome, from chronic states such as HF and atherosclerosis [91]. Targeted blockade of the IL-1 pathway by colchicine and canakinumab is necessary in these cases for the effective reduction in the threat from acute events and the long-term risk of HF [90,91].

### 7.2. Clonal Hematopoiesis and Genetic Risk Stratification

In defining high-risk inflammatory phenotypes, a major role is played by recognition of CHIP. Mutations in genes (*DNMT3A*, *TET2*, and ASXL transcriptional regulator 1 (*ASXL1*)) affect the age-related expansion of somatic blood cells, which characterizes CHIP. These mutations affect the increase in all-cause mortality. Furthermore, they contribute to the increase in inflammation in the body. Patients with STEMI and the presence of CHIP mutations and hs-CRP > 5.8 mg/L have a significantly increased risk of death. This occurs because *TET2* and *ASXL1* mutations are associated with increased susceptibility of the atherosclerotic plaque to rupture [85]. It was also noted that inherited pathogenic variants in the telomerase reverse transcriptase (*TERT*) and *TET2* gene loci double the risk of MACE and are associated with metabolic disorders. This shows that epigenetic regulation plays a key role in linking genetic predispositions and environmental factors [86]. Scientific research suggests that CHIP carriers may derive greater benefits from intensive statin therapy and ticagrelor compared to individuals without these mutations. This shows that CHIP can be a potential biomarker for targeted cardiovascular interventions [85,86]. However, the routine clinical use of CHIP screening is premature due to practical barriers. Currently, genetic testing faces high costs, while its prolonged turnaround time limits its practicality in acute cases like ACS. Moreover, there is an absence of validated CHIP-guided treatment algorithms. Further clinical studies are needed to create a strong evidence base, which will enable the development of appropriate clinical guidelines in this field.

### 7.3. Genetics, Matrix Degradation, Lipid Metabolism and Cytokines

The individual degree of risk for ACS is defined by inherited polymorphism in genes that regulate matrix stability, systemic inflammation, and lipid metabolism. The balance between proteolytic activity and tissue inhibitors (TIMPs) can be disrupted by MMPs, especially MMP-3 (5A/6A alleles) and MMP-9 (C1562T). This leads to extracellular matrix destruction and thinning of the fibrous cap. This affects cardiac remodeling and increases the risk of atherosclerotic plaque damage, especially in young STEMI patients [92]. Genetic determinants of lipid biomarkers, such as polymorphism in Apo A1 (75G/A) and PON1 (Q192R), affect the premature development of CAD by enhancing atherogenesis. This happens even in the absence of other risk factors [93]. Specific single-nucleotide polymorphisms (SNPs) also significantly influence the pro-inflammatory environment. IL-6 (−174G>C) and tumor necrosis factor-alpha (TNF-α) (−308G>A) variants promote atherosclerotic plaque destabilization and impair myocardial contractility. The ethnic origin of patients (specifically Asian and Arab populations) also influences these effects [94]. The synthesis of these various genetic profiles is important for precision medicine and increasing and individualizing cardiological care [93,94].

### 7.4. SGLT2 Inhibitors and Inflammation

Emerging preclinical and observational data suggest that beyond metabolic action, SGLT2i may also have anti-inflammatory properties. In experimental models, empagliflozin and dapagliflozin suppress the NLRP3 inflammasome by alleviating mitochondrial dysfunction and reducing ROS production. This translates directly into limiting the release of pro-inflammatory IL-1β and IL-18 [95]. This mechanism could be particularly important in the case of patients with type 2 diabetes (T2DM) who undergo AMI. In them, concurrent kidney and heart damage significantly increases the risk of tissue damage and death [96]. The latest data indicate that the appropriate timing of SGLT2i initiation might be crucial in treatment efficacy. Early therapy initiation, even before discharging the patient from the hospital, was associated with lower hs-CRP and NLR levels and also delays the macrophage senescence process. Later therapy initiation (90 days after AMI) may result in a clear limitation of its benefits. In these patients, a persistent increase in IL-6 level was visible [87]. However, these promising mechanistic insights and biomarker reductions did not show the expected clinical benefits in large-scale trials. In the EMPACT-MI trial, early empagliflozin treatment did not lower the risk of a composite primary endpoint event, which was a first hospitalization for HF or death from any cause among patients presenting with an AMI and an increased risk of HF [88]. These findings underscore that while early SGLT2i application remains superior for NLRP3 pathway modulation and limiting RIR [95,96], translating these effects into a reduction in clinical endpoints in the acute post-MI phase remains a major challenge. Additionally, adequately powered, prospective RCTs are strictly required to definitively clarify the anti-inflammatory efficacy of SGLT2i.

### 7.5. Dynamics and Types of Immune Response

For the use of immunomodulatory treatment in ACS, it is important to understand that it is a very dynamic state. The period immediately after infarction starts with an initial inflammatory phase (1–4 days), which is dominated by M1 macrophages and Th1 lymphocytes. Then we have the reparative phase (days 3–14), during which M2 macrophages and T-regulatory lymphocytes (Treg) support tissue healing and scarring, in which fibroblasts are also involved. Studies show that peripheral blood mononuclear cells (PBMCs) during the first 48 h after infarction exhibit upregulation of the P2Y11 receptor (P2Y11R) and genes such as signal transducer and activator of transcription 3 (*STAT3*) and heme oxygenase 1 (*HMOX1*). They are responsible for the regulation of dendritic cells and polarization of immune cells [97]. This shows that we must distinguish chronic inflammation, which leads to unfavorable remodeling and increased residual inflammatory risk, from the protective inflammatory response, which is essential for removing necrotic tissues and repairing the myocardium [98]. The goal of precision medicine in the future should be the use of advanced immune system profiling systems in order to treat the excessive inflammatory response without endangering the necessary reparative pathways, ensuring heart regeneration [97,98]. We summarize the recommended timing windows, clinical contexts and pathophysiological rationales for each anti-inflammatory agent in Table 4.

## 8. Discussion

### 8.1. The Superiority of Precision Immunomodulation

The new approach to treatment in cardiology favors a transition from non-specific immunosuppressive treatment to targeted immunomodulation. Clinical evidence collected in this review confirms the truth of the inflammatory hypothesis claims. Treatment targeted at specific inflammatory pathways, such as NLRP3/IL-1β/IL-6, is an effective method of reducing RIR. In one of the recent meta-analyses [90], immunomodulatory drugs as a group did not show a statistically significant reduction in MACE (*p* = 0.09). The lack of unambiguous results regarding the effectiveness of this type of therapy shows that inflammation in coronary heart disease (CHD) should not be treated with a random anti-inflammatory drug. This is also clearly demonstrated in the example of the CIRT trial [17] regarding methotrexate. Despite its important role in the treatment of systemic autoimmune diseases, it did not reduce the levels of IL-6 and hs-CRP. Pathophysiologically, this is of key importance because IL-6 stimulates hepatocytes in the liver to synthesize CRP. Failure to inhibit this pathway leads to further damage to the coronary vessel wall through inflammatory signals from the NLRP3 inflammasome. This, therefore, translates into a lack of satisfactory cardiovascular protection. The results of CIRT contrast with the success of the CANTOS [4] and COLCOT [14] trials. There, the degree of therapeutic benefits achieved was directly proportional to the reduction in pro-inflammatory IL-6. These findings suggest that the effectiveness of the implemented therapy does not depend on the strength of the immunosuppressive drug’s action itself, but rather on the precision in reducing pro-thrombotic inflammatory pathways of the NLRP3 inflammasome.

### 8.2. Impact of Clinical Trial Results on Clinical Practice

The consequences of the results of these clinical trials are recent changes introduced by the FDA and Health Canada. These organizations accepted the inclusion of colchicine in therapy as prevention in adult patients with CHD to reduce cardiovascular risk [58]. The ongoing changes indicate a growing awareness of the existence of RIR, which occurs even in patients optimally treated with statin therapy and achieving LDL-C therapeutic targets. In some of these patients, chronic inflammation persists, characterized by high hs-CRP values. This shows that lipid reduction alone, although necessary, does not completely inhibit all processes of atherosclerosis progression. Unfortunately, there is still a very large difference between the content of guidelines and clinical practice. In the analysis of colchicine prescribing in the USA, only a slight increase in its share in therapy has been noted since the publication of the LoDoCo2 and COLCOT trials [73]. This is caused by practical limitations and safety concerns in routine care. Long-term treatment adherence is frequently challenged by GI intolerance, particularly diarrhea, which is a primary reason for drug discontinuation [14,48]. Furthermore, colchicine has a narrow therapeutic window and an increased risk of toxicity in patients with renal dysfunction. Colchicine also requires caution regarding potential drug interactions with statins or strong CYP3A4 inhibitors [101]. While the risk of severe infections is a known concern, findings from the LoDoCo2 trial raised questions regarding a non-significant numerical increase in non-cardiovascular mortality [48]. Nevertheless, this cautious implementation significantly impacts public health costs. It is estimated that actively including colchicine in the treatment of appropriate patients with stable CAD would result in a reduction in the incidence of MACE by 226,000 in the United States alone within 3 years [73]. Experts predict that lowering LDL-C, along with inhibiting inflammation, will become the standard of care for patients with atherosclerosis in the future [58]. The above suggests that currently, we no longer need more evidence of the effectiveness of anti-inflammatory therapy. Instead, we need appropriate tools to assess the risk of its initiation and the selection of patients who will benefit from it. Key to this approach is the use of biomarkers through which we will identify these patients and minimize the risk of adverse effects.

### 8.3. Genetics in Risk Assessment

One of the latest discoveries described in this work is the role of CHIP as a mediator of high inflammatory risk. The presence of pathogenic variants in the *TET2* or *ASXL1* genes leads to macrophage hyperreactivity [85]. Such patients have a completely different inflammatory phenotype. In clinical practice, this is visible if CHIP mutations and elevated hs-CRP levels are present in STEMI patients. These patients then have a significantly higher risk of death than those without the present mutations. This results from the increased sensitivity of the atherosclerotic plaque, which is more susceptible to fibrous cap ruptures [85]. This suggests the possibility of using CHIP mutation screening in precision cardiology to identify patients in a particularly high-risk group. Furthermore, data indicate that these patients may derive greater benefits from intensive statin therapy and ticagrelor [85]. This is potentially a tangible example of how genetic information could influence the selection of the most beneficial pharmacological treatment.

### 8.4. Optimal Timing of Treatment

One of the most important aspects of precision immunomodulatory treatment is the appropriate moment of its initiation. The early immune response after MI is responsible for the removal of necrotic tissue, while during the later phase, scar maturation occurs in the myocardium [97]. It should also be remembered that in ACS, inflammation can perform good functions by helping in myocardial repair, but can also have a negative impact and lead to fibrosis and HF. It follows, therefore, that inappropriate anti-inflammatory treatment could paradoxically impair the formation of a stable scar, increasing the risk of cardiac wall rupture [98]. Adverse inflammation is characterized by overactivation of the NLRP3 inflammasome and dominates in the acute phase. It is this that constitutes the main goal of effective anti-inflammatory pharmacotherapy. The study by Cliff et al. [87] shows that the benefits of inhibiting NLRP3 and macrophage senescence with empagliflozin are time-dependent. Early initiation of therapy, even before hospital discharge, significantly lowers NLR and the hs-CRP level. In contrast, a delay in treatment by 90 days results in the loss of these anti-inflammatory abilities. The key importance of proper timing is also visible in the example of the P2Y11R in PBMCs. Its highest expression is in the therapeutic window of the first 48 h after MI, which is the time when the heart is most susceptible to repair signals [97]. This indicates that therapies targeted at this receptor should be initiated precisely then. An attempt to modulate this receptor at a later time would be ineffective due to the lack of a binding point for the drug. The above data demonstrate that equally important to exactly what we treat is when exactly we initiate the appropriate treatment.

### 8.5. Limitations and Future Directions

Despite promising evidence, several limitations must be addressed. Most major trials have been conducted in Western populations. Cytokine polymorphisms (IL-6 and TNF-α) show significant ethnic variability [94]. Future research should focus on this variability to provide evidence on which biomarkers will ultimately be the most appropriate as risk indicators so they can be used in a uniform system. In this context, evidence from the PRAISE registry shows that 1-year outcomes are largely similar between STEMI and NSTEMI patients [102]. Differences in reinfarction risk appear to be caused by baseline patient characteristics and treatment patterns rather than the infarct type itself [102]. This is relevant for targeted immunomodulation, as it supports the idea that identifying individual high-risk phenotypes is more crucial for long-term prognosis than relying on the traditional ischemic classification. It should also be a priority to determine the impact long-term interleukin blockade has on patients. Time intervals for initiating appropriate therapies should also be definitively established, and the creation of screening protocols for CHIP mutation testing should be considered. The future care for patients with ACS will likely consist of systems integrating the above assumptions to make treatment as precise as possible.

## 9. Conclusions

Modern precision cardiology requires the implementation of immunomodulatory therapies for the NLRP3/IL-1β/IL-6 pathway. Effective RIR reduction relies on choosing the appropriate therapy target, personalization based on genetic profiling (CHIP mutations), and strict adherence to therapeutic windows. The evolution of ACS treatment will likely depend on the synergy between established lipid level control and targeted immunomodulation. Integration of advanced immunological methods and genetic screening into routine clinical practice appears to be a promising future perspective. However, this transition from research into standardized practice requires further large-scale validation for maximization of clinical benefits and reduction in the risk of adverse effects.

## Figures and Tables

**Table 1:** Summary of major clinical trials evaluating anti-inflammatory therapies in cardiovascular disease.

Trial	Drug	Primary Target	Patient Population	Key Clinical Outcomes
CANTOS [4]	Canakinumab	IL-1β	Post-MI with hs-CRP > 2 mg/L	15% reduction in MACE; significant lowering of hs-CRP and IL-6; small increase in fatal infections.
LoDoCo2 [48]	Colchicine	NLRP3 inflammasome	Stable chronic CAD	31% relative risk reduction in CV death, MI, or stroke; high rate of GI-related discontinuation.
CIRT [17]	Methotrexat	Non-specific	Previous MI or multivessel CAD + T2DM/metabolic syndrome + low baseline hs-CRP (median 1.6 mg/L)	Neutral results; no reduction in MACE, hs-CRP, or IL-6.
COLCOT [14]	Colchicine	NLRP3 inflammasome	Acute phase post-MI (<30 days)	Significant reduction in serious CV complications; reduction in hs-CRP levels.
CLEAR-SYNERGY [16]	Colchicine	NLRP3 inflammasome	Acute MI + PCI	Neutral results; lowered CRP but did not show a statistically significant reduction in MACE.
RESCUE [12,13]	Ziltivekimab	IL-6	CKD with hs-CRP > 2 mg/L	hs-CRP reduced by 88–92%; reduction in NLR; increased triglycerides observed.

**Table 2:** Summary of drug dosages and administration schedules in major anti-inflammatory clinical trials.

Trial	Drug	Dosing Regimen in Trial *
CANTOS [4]	Canakinumab	50 mg, 150 mg, or 300 mg s.c. every 3 months
LoDoCo2 [48]	Colchicine	0.5 mg once daily
CIRT [17]	Methotrexate	15–20 mg weekly
COLCOT [14]	Colchicine	0.5 mg once daily
CLEAR-SYNERGY [16]	Colchicine (+Spironolactone **)	Initial: 0.5 mg twice daily (if ≥70 kg) or once daily (if <70 kg) for 90 days, then 0.5 mg once daily Modified: 0.5 mg once daily for all patients regardless of weight or treatment duration.
RESCUE [12,13]	Ziltivekimab	7.5 mg, 15 mg, or 30 mg s.c. every 4 weeks

* Matching placebo regimens were administered in all trials. ** Spironolactone was administered as part of the combined therapy protocol in the CLEAR-SYNERGY trial.

**Table 3:** Patient stratification and targeted anti-inflammatory frameworks based on clinical, immunological and genetic phenotypes.

Patient Phenotype	Suggested Therapy *	Rationale and Clinical Evidence	References
Elevated hs-CRP	Colchicine	Acute ACS: targeted reduction in persistent RIR during the hyperacute phase. Chronic ACS: sustained suppression of NLRP3 inflammasome activation in stable atherosclerotic plaques. The CANTOS trial established the clinical framework for this phenotype, proving efficacy in patients with baseline hs-CRP > 2 mg/L.	[4,14,48]
	Ziltivekimab (Under clinical evaluation)	Directly targets the downstream IL-6 pathway, demonstrating profound and consistent reductions in baseline hs-CRP levels.	[12]
CKD	Canakinumab (Experimental) Ziltivekimab (Under clinical evaluation)	In chronic post-ACS or stable chronic CAD settings: direct targeting of the IL-1β/IL-6 atherogenic axis. Provides systemic anti-inflammatory effects and a significant reduction in hs-CRP without raising renal clearance or drug-toxicity concerns in patients with impaired eGFR.	[7,12,77]
Presence of CHIP Mutations **	Standard ACS therapies (Statins + Ticagrelor)	Somatic mutations (e.g., *TET2* and *ASXL1*) accelerate clonal hematopoiesis, driving severe myeloid hyperreactivity, systemic leukocytosis, and extreme plaque vulnerability. Represents a primary genetic high-risk phenotype. Targets for prospective long-term risk stratification post-stabilization.	[19,85,86]
High NLR or ALI Index	Ziltivekimab or Colchicine	Subacute to chronic phases suppress myeloid-driven systemic vascular inflammation. Effectively mitigates neutrophilia, high leukocyte homing and macrophage-mediated matrix degradation within advancing atherothrombotic plaques.	[13,41]
T2DM + Post-ACS	SGLT2i ***	Early post-discharge initiation provides multimodal cardioprotection by combining systemic renal–metabolic benefits with indirect anti-inflammatory actions. Dampens downstream NLRP3 activation, improves endothelial functionality, and attenuates adverse left ventricular remodeling.	[87,88,89]

* Specific oral NLRP3 inhibitors, such as dapansutrile, are currently excluded from this table as their evaluation remains confined to non-cardiovascular settings (e.g., acute gout flare), with no clinical outcome data yet available for post-ACS prevention. ** A comprehensive discussion of the underlying molecular mechanisms, genetic risk stratification, and specific clinical implications of CHIP is explicitly provided in Section 7.2. *** In the acute post-MI setting, clinical trial evidence (EMPACT-MI) shows that early in-hospital initiation of SGLT2i causes a significant reduction in HF hospitalizations during the vulnerable post-acute phase, rather than altering hard ischemic endpoints. The detailed molecular pathways of these metabolic interventions and related structural surrogate trials are further expanded in Section 7.4.

**Table 4:** Recommended timing windows and clinical contexts for anti-inflammatory therapies in coronary artery disease.

Agent *	Clinical Context	Optimal Timing Window	Pathophysiological and Clinical Rationale
Colchicine	STEMI	Immediate (within 24 h) of first medical contact or primary PCI	Suppresses the acute, intense inflammatory surge induced by cholesterol crystals during reperfusion. Early initiation aims to limit infarct size and reduce early ischemic recurrence [14].
	NSTEMI	Within 72 h of clinical stabilization	Targets microvascular inflammation and stabilizes high-risk non-culprit plaques to prevent early remodeling before hospital discharge [14].
	Stable CAD	Chronically, at least 30 days post-ACS (or in stable outpatients)	Provides long-term reduction in RIR. Supported by the LoDoCo2 trial, demonstrating a 31% reduction in MACE [48].
Anakinra (anti-IL-1)	STEMI	Ultra-early (within 12 h) of symptom onset	Aimed at blunting the acute IL-1 storm immediately following reperfusion to prevent adverse remodeling and subsequent HF [79,83].
	NSTEMI/ Stable CAD	Not recommended	Lack of clinical efficacy or pathophysiological basis for acute receptor blockade outside the hyperacute ischemic tissue injury phase [80,91].
Canakinumab (anti-IL-1β)	Stable CAD	Chronically (every 3 months), initiated >4–6 weeks post-ACS stabilization	Formulated to control persistent, low-grade systemic vascular inflammation. The CANTOS trial proved efficacy exclusively in stable patients with a baseline hs-CRP > 2 mg/L [4].
Ziltivekimab (anti-IL-6)	Stable CAD and CKD	Chronically (once monthly)	Specifically targets the downstream IL-6 atherogenic pathway, which is highly upregulated in patients with concomitant CKD [12].
SGLT2i **	STEMI/ NSTEMI	Early in-hospital (within 24–72 h) post-stabilization, before discharge	Reduces acute wall stress via osmotic diuresis/natriuresis, shifts myocardial metabolism to efficient ketone bodies, and decreases systemic NLRP3 activation during the vulnerable post-acute phase [87,88].
	Stable CAD	Chronically, as baseline therapy	Provides continuous cardioprotection by mitigating chronic low-grade inflammation, improving endothelial function, and preventing secondary HF hospitalization [95,96].
P2Y11R Modulators	STEMI/ NSTEMI	Hyperacute phase (within hours) during or immediately post-PCI	Extracellular ATP released from damaged myocardium activates endothelial and leukocyte P2Y11 receptors. Acute modulation stabilizes endothelial permeability and regulates the pro- vs. anti-inflammatory balance of macrophage activation [22,99].
	Stable CAD	Under investigation (chronic maintenance)	Targeted at preventing ATP-driven chronic endothelial dysfunction and leukocyte homing into advancing atherosclerotic plaques [44,100].

* Specific oral NLRP3 inhibitors, such as dapansutrile, are currently excluded from this table as their evaluation remains confined to non-cardiovascular settings (e.g., acute gout flare), with no clinical outcome data yet available for post-ACS prevention. ** In the acute post-MI setting, clinical trial evidence (EMPACT-MI) shows that early in-hospital initiation of SGLT2i causes a significant reduction in HF hospitalizations during the vulnerable post-acute phase, rather than altering hard ischemic endpoints.

## Data Availability

The original contributions presented in this study are included in the article. Further inquiries can be directed to the corresponding authors.

## Abbreviations

The following abbreviations are used in this manuscript:

ACS	Acute coronary syndromes
AKI	Acute kidney injury
ALI	Advanced lung cancer inflammation index
AMI	Acute myocardial infarction
ASC	Apoptosis-associated speck-like protein containing CARD
ASCVD	Atherosclerotic cardiovascular disease
*ASXL1*	ASXL transcriptional regulator 1
BMI	Body mass index
CAD	Coronary artery disease
CARD	Caspase recruitment domain
CCBs	Calcium-channel blockers
CCs	Cholesterol crystals
CHD	Coronary heart disease
CHIP	Clonal hematopoiesis of indeterminate potential
CKD	Chronic kidney disease
CRP	C-reactive protein
CVD	Cardiovascular disease
DAMPs	Damage-associated molecular patterns
*DNMT3A*	DNA methyltransferase 3 alpha
FDA	Food and Drug Administration
GI	Gastrointestinal
GSDMD	Gasdermin D
HDL-C	High-density lipoprotein cholesterol
HF	Heart failure
*HMOX1*	Heme oxygenase 1
hs-CRP	High-sensitivity C-reactive protein
IFN-γ	Interferon gamma
IL-18	Interleukin-18
IL-1α	Interleukin-1α
IL-1β	Interleukin-1β
IL-6	Interleukin-6
INR	International normalized ratio
IRF-1	Interferon regulatory factor 1
LDL-C	Low-density lipoprotein cholesterol
LOX-1	Lectin-like oxidized low-density lipoprotein receptor-1
LRRs	Leucine-rich repeats
LVEF	Left ventricular ejection fraction
MACEs	Major adverse cardiovascular events
MCP-1	Monocyte chemoattractant protein-1
MeSHs	Medical subject headings
MI	Myocardial infarction
MMPs	Matrix metalloproteinases
NACHT	Central NBD-containing ATPase domain
NF-kB	Nuclear factor-kappa B
NLR	Neutrophil-to-lymphocyte ratio
NLRP3	NLR family pyrin domain containing 3
NLRs	NOD-like receptors
NSTEMI	Non-ST-elevation myocardial infarction
ox-LDL	Oxidized low-density lipoprotein
P2Y11R	P2Y11 receptor
PAI-1	Plasminogen activator inhibitor-1
PAMPs	Pathogen-associated molecular patterns
PBMCs	Peripheral blood mononuclear cells
PCI	Percutaneous coronary intervention
PRR	Pattern recognition receptors
PYD	Pyrin domain
QALY	Quality-adjusted life year
RCTs	Randomized controlled trials
RIR	Residual inflammatory risk
ROS	Reactive oxygen species
SGLT2is	SGLT2 inhibitors
SNPs	Single-nucleotide polymorphisms
*STAT3*	Signal transducer and activator of transcription 3
STEMI	ST-elevation myocardial infarction
T2DM	Type 2 diabetes mellitus
*TERT*	Telomerase reverse transcriptase
*TET2*	Ten-eleven translocation 2
TIMPs	Tissue inhibitors of metalloproteinases
TLR	Toll-like receptors
TNF-α	Tumor necrosis factor-alpha
Treg	T-regulatory lymphocytes
VCAM-1	Vascular cell adhesion molecule-1
VKA	Vitamin K antagonist
